# Application of Silica Nanoparticles Improved the Growth, Yield, and Grain Quality of Two Salt-Tolerant Rice Varieties under Saline Irrigation

**DOI:** 10.3390/plants13172452

**Published:** 2024-09-02

**Authors:** Wenyu Jin, Lin Li, Wenli He, Zhongwei Wei

**Affiliations:** 1 College of Breeding and Multiplication (Sanya Institute of Breeding and Multiplication), Hainan University, Sanya 572000, China; jwy825@126.com (W.J.); hewenli43@163.com (W.H.); 2 National Center of Technology Innovation for Saline-Alkali Tolerant Rice in Sanya, Sanya 572024, China; 3 State Key Laboratory of Hybrid Rice, Hunan Hybrid Rice Research Center, Changsha 410125, China

**Keywords:** antioxidant enzyme, salt stress, SiO_2_ NPs, rice, RVA characteristics

## Abstract

Salt stress significantly reduces rice yield and quality and is a global challenge, especially in arid and semi-arid regions with limited freshwater resources. The present study was therefore conducted to examine the potential of silica nanoparticles (SiO_2_ NPs) in mitigating the adverse effects of saline irrigation water in salt-tolerant rice. Two salt-tolerant rice varieties, i.e., Y liangyou 957 (YLY957) and Jingliangyou 534 (JLY534), were irrigated with 0.6% salt solution to simulate high-salt stress and two SiO_2_ NPs were applied, i.e., control (CK) and SiO_2_ NPs (15 kg hm^−2^). The results demonstrated that the application of SiO_2_ NPs increased, by 33.3% and 23.3%, the yield of YLY957 and JLY534, respectively, compared with CK, which was primarily attributed to an increase in the number of grains per panicle and the grain-filling rate. Furthermore, the application of SiO_2_ NPs resulted in a notable enhancement in the chlorophyll content, leaf area index, and dry matter accumulation, accompanied by a pronounced stimulation of root system growth and development. Additionally, the SiO_2_ NPs also improved the antioxidant enzyme activities, i.e., superoxide dismutase, peroxidase, and catalase activity and reduced the malondialdehyde content. The SiO_2_ NPs treatment effectively improved the processing quality, appearance quality, and taste quality of the rice. Furthermore, the SiO_2_ NPs resulted in improvements to the rapid viscosity analyzer (RVA) pasting profile, including an increase in peak viscosity and breakdown values and a reduction in setback viscosity. The application of SiO_2_ NPs also resulted in a reduction in crystallinity and pasting temperature owing to a reduction in the proportion of B2 + B3 amylopectin chains. Overall, the application of silica nanoparticles improved the quality of rice yield under high-salt stress.

## 1. Introduction

Salinity is a significant global challenge impacting agricultural productivity, water quality, and soil health [1,2]. Approximately 954 million hectares, or 20% of the irrigated land, are affected by salinization worldwide; 99 million hectares of land are affected by salinization in China [3]. The soil salinization is becoming increasingly severe, leading to a substantial reduction in crop yields and posing a formidable challenge to global food security and agricultural sustainability [4,5]. Therefore, exploring effective methods to mitigate high-salinity stress and to enhance crop resilience has become crucial to ensure food security.

High-salt stress generally refers to an environment that inhibits the normal growth of rice and seriously reduces the rice yield [3,6]. High-salt stress leads to many adverse effects, including root growth retardation, leaf curling, reduced plant height, reduced number of tillers, reduced number of spikelets per spike, reduced grain filling rate, reduced thousand-grain weight, and ultimately, deterioration of rice quality and yield [7]. It has been shown that some rice cultivars can maintain their yield with the application of 0.3% saline irrigation water [8]; nevertheless, when the salt concentration exceeds 0.5%, the yield of even salt-tolerant rice varieties was substantially reduced [9]. However, the reduction in yield of salt-tolerant rice varieties under conditions of high-salt stress was less pronounced than that observed in salt-sensitive rice varieties. It is therefore of great importance to cultivate salt-tolerant rice varieties in order to expand the production of saline-alkali land. However, Li et al. [10] observed that under conditions of high-salt stress, the yield reduction of salt-tolerant rice varieties remained considerable. Consequently, the corresponding agronomic cultivation measures should also be implemented concurrently.

Silicon (Si) is recognized as an essential plant nutrient and has gained renowned attention owing to its involvement in regulating plant growth, development, and stress responses through various mechanisms [11,12,13]. Previous studies have indicated that Si fertilizers can promote root development, activity of antioxidant enzymes, and the stability of cell membranes, thereby alleviating the inhibitory effects of salt stress on rice [14,15]. However, these studies have primarily focused on the effects of traditional silicon fertilizers on conventional rice varieties, with relatively less research on nano-scale silicon.

Silica nanoparticles (SiO_2_ NPs), due to their unique nano-scale effects and surface characteristics, may exhibit higher absorption, translocation, and utilization efficiency within plants compared with traditional Si fertilizers [14,15]. Generally, SiO_2_ NPs can enter plant tissues more rapidly, tightly bind with biological membranes, and form a more stable protective layer that helps in further enhancing the rice tolerance against salt stress [14]. Additionally, SiO_2_ NPs may also improve the stress resistance and rice yield by regulating hormonal balance and promoting nutrient absorption and utilization in the plants [16]. However, a thorough exploration of the mechanisms and economic benefits of SiO_2_ NPs in alleviating salt stress and improving yield and quality traits over conventional Si fertilizer is still lacking.

The development of salt-tolerant rice with the ability to maintain yield is an important achievement in the scenario of saline agriculture [5,17]. Nevertheless, the growth, yield, and grain quality of salt-tolerant rice are also substantially affected under high-salt stress conditions [10,18]. Although salt-tolerance mechanisms in rice have been explored, there has been limited research on how SiO_2_ NPs improve rice growth and yield in saline environments. Therefore, the present study aimed to assess the effects of the application of SiO_2_ NPs on the growth, yield, and grain quality of salt-tolerant rice varieties under higher saline conditions.

## 2. Materials and Methods

### 2.1. Experimental Details

The experiment was conducted at the experimental base of National Saline-alkali Tolerant Rice Technology Innovation Center, Leyi Village, Hainan Province, China (18°26′00″ N, 108°54′00″ E) from January to May 2023. The region is a tropical marine climate, with an average annual temperature of 20–26 °C and an average annual precipitation of 1653.4 mm. The experimental field soil was sandy loam soil. The soil organic matter, total nitrogen, available phosphorus (P), and available potassium (K) were 18.6 g kg^−1^, 0.96 g kg^−1^, 42.6 mg kg^−1^, and 241.5 mg kg^−1^, respectively. Two approved high-yielding salt-tolerant rice varieties, i.e., Y liangyou 957 (YLY957) and Jingliangyou 534 (JLY534), were obtained from Hunan Hybrid Rice Research Center. The selection of both varieties as ‘salt tolerant’ is based on the reports of Jin et al. [18]. Seeds were sown on 10 January 2023, and 30-day-old seedlings were transplanted for manual single seedling transplanting with 30 × 15 cm plant spacing. Damaged and poor seedlings were replaced three days after transplanting to ensure better stand establishment. Pest, disease, and weed control measures were conducted in accordance with local high-yield rice cultivation practices and the guidelines of the provincial government.

### 2.2. Experimental Design and Treatments

The experiment was conducted using a randomized block design with two treatments, i.e., control (CK) with no exogenous SiO_2_ NPs application and a recommended application rate of 15 kg SiO_2_ NPs per hm^2^ based on our preliminary trials. The particle size of SiO_2_ nanoparticles was 30 ± 5 nm, the specific surface area was 600–800 m^2^ g^−1^, and the purity was 99.5%. The SiO_2_ NPs were commercial product purchased from Shanghai Maclin Biochemical Technology Co., Ltd. (Shanghai, China). The fertilizer was applied at 210 kg N hm^−2^ (as urea), 105 kg P_2_O_5_ hm^−2^ (as calcium magnesium phosphate), and 210 kg K_2_O hm^−2^ (as potassium chloride) per hm^2^. Nitrogen fertilizer was applied with a ratio of 4:3:3 as basal, tillering, and panicle fertilizers, respectively, whereas phosphatic fertilizer was applied as a one-time basal application. The potassium fertilizer was split, with 50% as basal and the remaining 50% at the panicle stage. The SiO_2_ NPs were applied as basal fertilizer mixed with all fertilizer, and then as a broadcasted treatment before transplanting the seedlings. Ridges (50 cm) were built between treatments and wrapped with plastic film to prevent cross-fertilization and water seepage. Each plot had an area of 30 m^2^. After transplanting, the seedlings were initially irrigated with fresh water to promote stand establishment. Seven days after transplanting, a mixture of seawater and freshwater adjusted to a 0.6% salinity concentration was used for irrigation for each treatment. The seawater and underground freshwater were, respectively, extracted by pumps and blended to 0.6% in salt ponds, and then piped to the fields. Then, the salinity was measured using portable conductivity meter (2266FS, Spectrum, WI, USA). The irrigation water was drained every three days and re-applied, with an additional drainage after rainy days. A water layer of 5 cm was maintained throughout the growing season, and irrigation was stopped seven days before harvest. In previous experiments, we applied SiO_2_ NPs at varying gradients and found that a rate of 15 kg hm^2^ yielded the best results in terms of yield. Rice yields can still be maintained despite application of more than 15 kg hm^2^ compared with CK treatment. However, due to the additional expenditure caused by the use of SiO_2_ NPs, the increased production is not enough to offset the SiO_2_ NPs, so we chose 15 kg hm^2^ SiO_2_ NPs to study the mechanism of the increase in yield and quality.

### 2.3. Sampling and Data Collection

#### 2.3.1. Agronomic Traits

At the middle tillering (MT), panicle initiation (PI), heading stage (HS), and maturity stage (MS), five uniform plants from each treatment were harvested; separated into roots, leaf sheaths, leaves, and panicles (post-emergence); oven dried at 80 °C; and weighed. The leaf area index was measured at the MT, PI, HS, and MS. The single leaf area (cm^2^) was calculated as leaf length (cm) multiplied by leaf width (cm) and then by 0.75. The leaf area index was determined by plot area. At MT, PI, HS, and MS, the SPAD values were measured using a portable chlorophyll meter (SPAD-502 PLUS). Five uniform plants from each replicate were selected, and six SPAD readings were taken at the midpoint of the sword leaf, one half to one third from the leaf tip, and averaged. At the HS, fresh rice root samples were cut, rinsed with deionized water, and soaked in water. Root morphological indices such as root length, root surface area, number of roots, and root average diameter were measured using a root scanner (GXY-A, Top Instrument, Hangzhou, China) and analyzed [19].

#### 2.3.2. Determination of Sodium (Na^+^) and Potassium Ions (K^+^)

Dry plant samples (0.5 g) in powder form were digested, and the Na^+^ and K^+^ content was then determined using a flame photometer [10].

#### 2.3.3. Malondialdehyde Content and Antioxidant Enzyme Activity

During the MT, PI, and HS, the top leaf of the rice plants from each treatment were immersed in liquid nitrogen and stored at −80 °C to determine the leaf malondialdehyde (MDA) and antioxidant enzyme activities such as superoxide dismutase (SOD), peroxidase (POD), and catalase (CAT) by following the instructions of the assay kit produced by Beijing Solaibao Technology Co., Ltd. (Beijing, China).

#### 2.3.4. Yield and Yield Attributes

At the MS, 15 hill plants were investigated at each plot to determine the effective panicle. On the basis of the average number of effective panicles, five uniform panicles were taken as one sample to measure the total number of grains per panicle, the number of filled grains per panicle, and the thousand-grain weight. In each plot, 200 hills were harvested, threshed, and cleaned, and yield was determined at 13.5% grain moisture content [20].

#### 2.3.5. Determination of Rice Quality

The grains were threshed manually, air-dried, and stored at room temperature for three months for grain quality attributes. Rice grain (200 g) from the stored grain lot was processed through rice huller (Thu35c, Satake, Hiroshima, Japan) and Jingmi rice grader (Zhejiang Top Cloud-Agri Technology Co., Ltd., Hangzhou, China) to obtain the milled rice and sieved (10 mesh) to remove the broken rice grains [21]. The head rice samples (50 g) were taken to determine the taste value by using a rice taste meter (STA1A, Satake, Hiroshima, Japan). Gel consistency and amylose starch contents were determined according to the agricultural industry standard of China NY/T 83-2017 and NY/T 2639, respectively [22]. The protein content was determined according to Li et al. [7]. Total starch content was determined using a total starch detection kit (Megazyme, K-TSTA, Beijing, China).

For determination of grain starch and amylopectin, the harvested grains were stored at 4 °C. Starch pasting and thermal properties were measured according to Yao et al. [22]. Starch pasting properties were analyzed by using a rapid viscosity analyzer (RVA) (RVA Super 4, Newport Scientific, Warriewood, NSW, Australia) and the thermal properties were measured using a differential scanning calorimeter (Q2000, Newport Scientific, Warriewood, NSW, Australia).

#### 2.3.6. Amylopectin Chain Length and X-ray Diffraction Analysis

The starch chain length distribution was determined by an ICS5000 ion chromatography system (ICS-5000, Thermo Fisher Scientific, Sunnyvale, CA, USA). The X-ray diffraction (XRD) analysis of starch was performed using an X’Pert Pro X-ray diffractometer (PANalytical, Almelo, The Netherlands). Starch samples were scanned at 4°/min over a 2θ range of 4–60° in steps of 0.02°. The scan duration was 10 min/sample.

### 2.4. Data Analysis

Analysis of variance (ANOVA) was conducted on the collected data using SPSS 19.0 software (SPSS, Inc., Chicago, IL, USA). The differences among the treatment means were separated using the least significant difference (LSD) test at a significance level of 0.05. All graphs were created using Origin 9.0 (OriginLab Corp., Northampton, MA, USA).

## 3. Results

### 3.1. Effects of SiO_2_ NPs Application on Rice Yield and Yield Components

The application of SiO_2_ NPs substantially affected the yield and yield components of the rice (Table 1). For example, the number of grains per panicle for YLY957 and JLY534 was increased by 10.6% and 11.1%, respectively, with SiO_2_ NPs applied, whereas no significant difference between the CK and SiO_2_ NPs treatment was noticed in terms of effective panicle number and thousand-grain weight for both rice varieties. However, SiO_2_ NPs application increased the grain filling rate in YLY957 by 8.2% and in JLY534 by 7.6% compared with CK. The SiO_2_ NPs increased the yield up to 33.3% and 23.3% of YLY957 and JLY534, respectively. The improvement in the number of grains per panicle and grain filling ultimately led to an increase in the overall yield of both salt-tolerant rice varieties.

### 3.2. Effects of SiO_2_ NPs Application on Agronomic Traits

**Figure 1:**
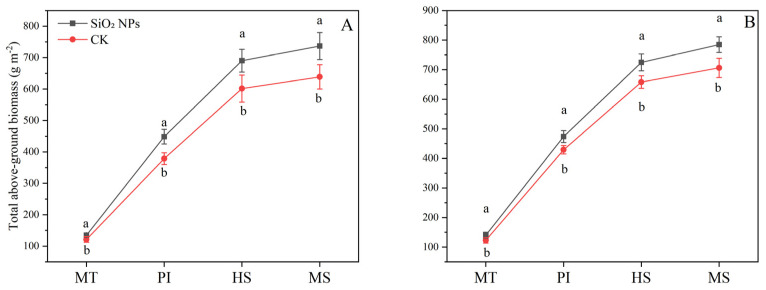
Effect of SiO_2_ NPs application on above-ground dry matter accumulation for Y liangyou 957 (**A**) and Jingliangyou 534 (**B**) under higher saline conditions. Values ± SD (n = 3) of the same cultivar with different letters are significantly different (*p* < 0.05). (**A**) Y liangyou 957; (**B**) Jingliangyou 534; CK, control; SiO_2_ NPs, silica nanoparticles; MT, middle tillering; PI, panicle initiation; HS, heading stage; MS, maturity stage.

The application of SiO_2_ NPs led to an increase in the above-ground dry biomass in both the varieties (Figure 1). Specifically, compared with CK, the application of SiO_2_ NPs resulted in an increase of 11.2% and 14.0% at MT, 18.5% and 10.4% at PI, 14.8% and 10.1% at HS, and 15.4% and 11.2% at MS in the above-ground dry biomass of YLY957 and JLY534, respectively. It is noteworthy that the enhancement in above-ground dry biomass of YLY957 was more pronounced than that in JLY534 across all growth stages.

**Figure 2:**
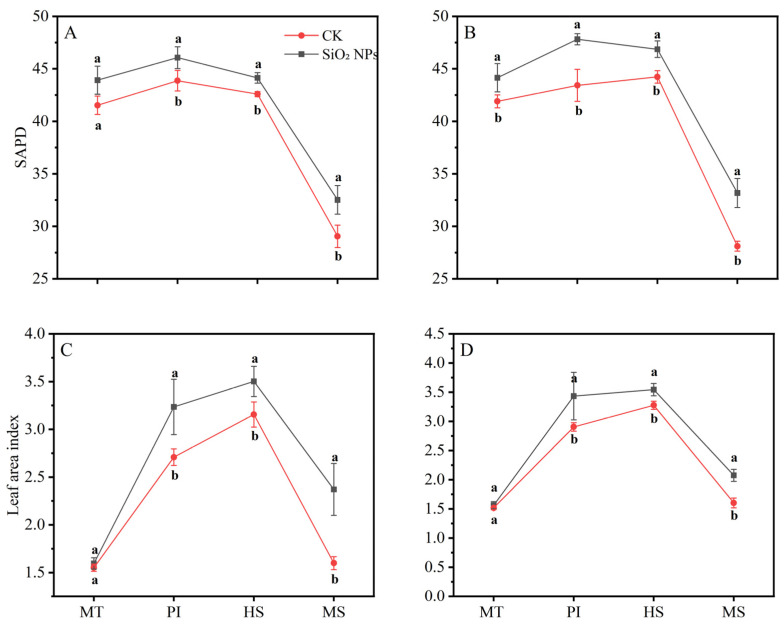
Effects of SiO_2_ NPs application on SPAD and leaf area index for Y liangyou 957 (**A**,**C**) and Jingliangyou 534 (**B**,**D**) under higher saline conditions. Values ± SD (n = 3) of the same cultivar with different letters are significantly different (*p* < 0.05). (**A**,**C**) Y liangyou 957; (**B**,**D**) Jingliangyou 534; CK, control; SiO_2_ NPs, silica nanoparticles; MT, middle tillering; PI, panicle initiation; HS, heading stage; MS, maturity stage.

**Figure 3:**
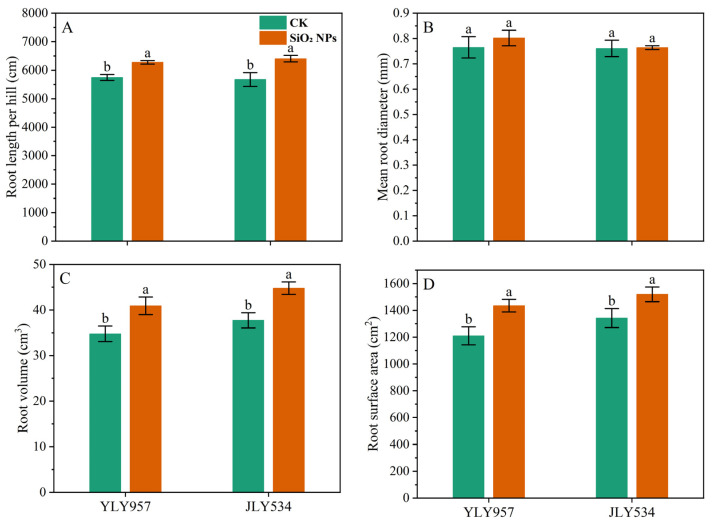
Effects of SiO_2_ NPs application on root morphological indices under higher saline conditions. Values ± SD (n = 3) of the same cultivar with different letters are significantly different (*p* < 0.05). (**A**,**C**) YLY957; (**B**,**D**) JLY534; CK, control; SiO_2_ NPs, silica nanoparticles; YLY957, Y liangyou 957; JLY534, Jingliangyou 534.

The LAI and SPAD values for both treatments exhibited a pattern of initial increase followed by a decrease as the growth cycle progressed (Figure 2). During the MT, no significant difference in the LAI was noticed between the treatments. However, from the PI to the MS, the LAI in both rice varieties applied with SiO_2_ NPs was significantly higher than that in the CK. In terms of LAI, the application of SiO_2_ NPs led to an increase of 5.7% and 5.4% for YLY957 and JLY534, respectively, at MT; of 5.0% and 10.1% at PI; of 3.6% and 6.0% at HS; and of 11.9% and 18.0% at MS. The decline in the LAI and SPAD values from the HS to the MS was less pronounced in the SiO_2_ NPs treatments than the CK. Furthermore, application of SiO_2_ NPs also improved the root morphology of both rice varieties (Figure 3). Specifically, the SiO_2_ NPs application increased the root length, root volume, and root surface area by 9.3%, 17.7%, and 18.6% in YLY957 and by 12.9%, 18.7%, and 13.2% in JLY534, respectively. However, no significant difference was noted for root diameter between the SiO_2_ NPs and the CK treatment for both varieties.

### 3.3. Effects of SiO_2_ NPs Application on Leaf Na^+^ and K^+^

**Figure 4:**
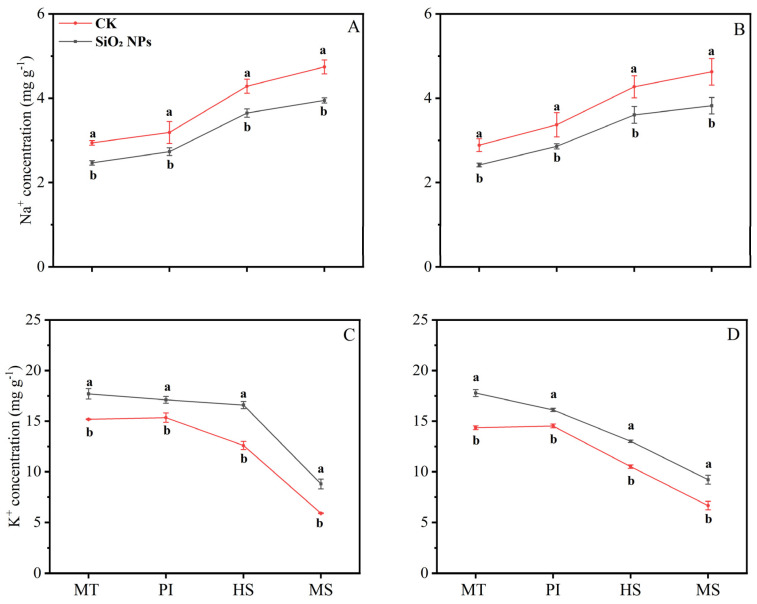
Effect of SiO_2_ NPs application on Na^+^ (sodium) and K^+^ (potassium) ion content in leaves for Y liangyou 957 (**A**,**C**) and Jingliangyou 534 (**B**,**D**) under higher saline conditions. Values ± SD (n = 3) of the same cultivar with different letters are significantly different (*p* < 0.05). (**A**,**C**) Y liangyou 957; (**B**,**D**) Jingliangyou 534; CK, control; SiO_2_ NPs, silica nanoparticles; MT, middle tillering; PI, panicle initiation; HS, heading stage; MS, maturity stage.

The application of SiO_2_ NPs altered the Na⁺ and K⁺ content in both rice varieties across various growth stages (Figure 4). Specifically, SiO_2_ NPs application led to a decrease in Na⁺ content at each sampling stage, while concurrently increasing the K⁺ content. Notably, from the MT to the MS, there was a progressive increase in Na⁺ content and a corresponding decrease in K⁺ content within the leaves of both rice varieties. At the MS, the reduction in leaf Na⁺ content was most pronounced, i.e., 16.7% for YLY957 and 17.4% for JLY534. Additionally, the K⁺ content at MS showed a significant increase, i.e., 49.3% and 38.3% for YLY957 and JLY534, respectively, that was the higher increase than the other stages.

### 3.4. Effects of SiO_2_ NPs Application on Leaf Antioxidant Enzymes Activity and MDA Content

**Figure 5:**
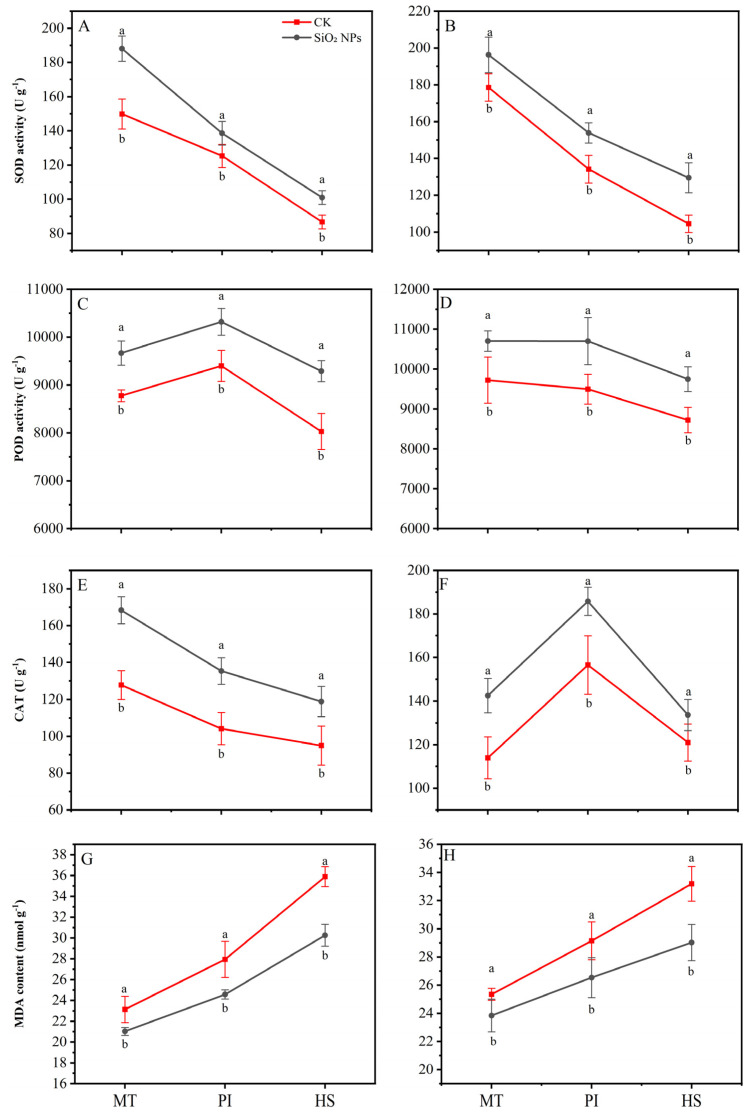
Effects of SiO_2_ NPs application on antioxidant oxidase activity and MDA content in leaves for Y liangyou 957 (**A**,**C**,**E**,**G**) and Jingliangyou 534 (**B**,**D**,**F**,**H**) under higher saline conditions. Values ± SD (n = 3) of the same cultivar with different letters are significantly different (*p* < 0.05). (**A**,**C**) Y liangyou 957; (**B**,**D**) Jingliangyou 534; CK, control; SiO_2_ NPs, silica nanoparticles; MT, middle tillering; PI, panicle initiation; HS, heading stage; MDA, malondialdehyde; SOD, superoxide dismutase; POD, peroxidase; CAT, catalase.

The application of SiO_2_ NPs substantially improved the activity of antioxidant enzymes, i.e., SOD, POD, and CAT, while reducing the content of MDA across all growth stages (Figure 5). For the YLY957, compared with the CK, the SiO_2_ NPs treatment exhibited the highest increase in SOD and CAT activities during the MT, with increases of 25.5% and 31.8%, respectively. The maximum increase, with 15.7% enhancement, was observed in POD activity at HS. For JLY534, the maximum CAT and POD activities were noticed at MT and HS, respectively. Notably, the lowest MDA content was noticed at HS for both rice varieties, with decreases of 18.6% for YLY957 and 14.4% for JLY534. Furthermore, the extent of MDA reduction varied across different stages, with maximum decrease at HS followed by the PI and the MT.

### 3.5. Effects of SiO_2_ NPs Application on Rice Quality

The application of SiO_2_ NPs improved the rice quality, especially the milling rice rate and head rice rate (Table 2). The brown rice rate was 2.2% and 1.0% higher in the SiO_2_ NPs treatment than CK for YLY957 and JLY534, respectively. In the YLY957, the milling and head rice rate was increased by 2.5% and 2.4%, respectively, with SiO_2_ NPs application compared with CK. In the JLY534, the milling rice rate and head rice rate was increased by 2.1% and 3.1%, respectively, with SiO_2_ NPs application. However, SiO_2_ NPs application reduced the chalkiness degree and the chalky grain rate while marginally increasing the length/width ratio.

Furthermore, the SiO_2_ NPs application substantially improved the total starch and amylose content and gel consistency of the both rice varieties and reduced the protein content significantly (Table 3). The total starch and amylose contents were increased by 3.3% and 14.8% (in YLY957) and 5.2% and 27.2% (in JLY534), respectively, with SiO_2_ NPs application compared with CK. Moreover, the protein content in YLY957 and JLY534 was decreased by 8.3% and 12.0%, respectively, whereas the gel consistency and alkali spreading value were increased by 6.8–9.4% and 3.0–17.1%, respectively, for both varieties with SiO_2_ NPs application compared with CK. Moreover, SiO_2_ NPs treatment significantly improved the eating value of both rice varieties.

### 3.6. Effects of SiO_2_ NPs Application on RVA and Gelatinization Characteristics

The SiO_2_ NPs had a significant impact on the RVA characteristics of both rice varieties (Table 4). The peak viscosity (PV), trough viscosity (TV), and breakdown viscosity (BD) were increased by 13.1%, 10.1%, and 20.7% (in YLY957) and 1.0%, 9.31%, and 18.3% (JLY534), respectively, whereas the consistence viscosity (CSV), setback viscosity (SB), and pasting temperature (PT) were decreased by 21.1%, 147.0%, and 0.2% (in YLY957) and 12.7%, 5.1%, and 2.1% (in JLY534), respectively, in the SiO_2_ NPs treatment compared with the CK. Overall, SiO_2_ NPs treatment substantially improved the quality characteristics of rice for both varieties, especially in improving viscosity and stability.

In addition, the SiO_2_ NPs treatment regulated the gelatinization characteristics of the rice, specifically by reducing the gelatinization enthalpy (Table 5). For YLY957, no significant difference was noticed in the onset temperature, peak temperature, or final temperature between the SiO_2_ NPs and CK treatments; however, the SiO_2_ NPs treatment significantly reduced the onset temperature, peak temperature, and final temperature, with a decrease of 2.4%, 1.7%, and 5.0%, respectively, in JLY534. In addition, the SiO_2_ NPs reduced the enthalpy value of YLY957 and JLY534 by 8.5% and 14.1%, respectively, compared with CK.

### 3.7. Effects of SiO_2_ NPs Application on Distribution of Amylopectin Chain Lengths

The SiO_2_ NPs treatment had a significant impact on the distribution of amylopectin chain lengths and crystallinity of the both rice varieties (Table 6). Compared with CK, the SiO_2_ NPs treatment reduced the relative crystallinity by 8.4% in the YLY957 and increased it by 7.5% in the JLY534. In addition, all samples had an A-type crystallization pattern, whereas the SiO_2_ NPs treatment did not change the crystallization pattern. Moreover, the SiO_2_ NPs treatment increased the DP 6–12 chain length (A) by 8.7% and 3.8% and increased the DP 13–24 (B1) by 2.1% and 3.3% in YLY957 and JLY534, respectively. Furthermore, the SiO_2_ NPs treatment significantly decreased the DP 25–36 (B2) and DP ≥ 37 (B3) of both rice varieties.

## 4. Discussion

Rice is a principal cereal crop among all food crops globally; however, it exhibits sensitivity to soil salinity [23]. Therefore, developing strategies to mitigate the impact of salinity on rice growth and productivity is important for global food security [11,24]. The advent and use of nanotechnology have opened new prospects for agriculture, with particular emphasis on the roles on nano-fertilizers for crop improvement under normal and stress conditions [11].

In our study, the increase in grain filling rate and grains per panicle are associated with the increased rice yield with the application of SiO_2_ NPs. The synergistic application of SiO_2_ NPs potentially alleviates the negative effects of soil salinity, with reduced Na+ absorption and improved photosynthetic rate, grain filling rate, and panicle weight [12]. In our previous study, the YLY957 and JLY534 varieties achieved yields of 9.9 and 9.6 t hm^−2^, respectively, under conditions of freshwater irrigation [25]. However, when subjected to 0.6% saline irrigation, the yield of these two varieties exhibited a marked decline, with reductions of 75.8% and 68.8%, respectively. While the application of SiO_2_ NPs has the potential to enhance rice yield, it remains comparatively low in comparison with that observed under freshwater irrigation. It is therefore imperative that further research be conducted on the application of salt-tolerant rice and SiO_2_ NPs in order to provide a foundation for the future enhancement of rice yield.

Our results show that the main reasons for the increased yield of SiO_2_ NPs are improved root growth and increased dry matter accumulation and leaf characteristics such as chlorophyll content, antioxidant enzyme activity, and K^+^ content (Figure 1, Figure 2, Figure 3, Figure 4 and Figure 5). The excessive accumulation of Na^+^, Cl^−^, and sulfate ions in plant roots disturbs osmotic potential, thus restricting water intake and plant growth, and in some cases, leading to plant mortality [11]. Improvements in root morphological traits may be due to the ability of SiO_2_ NPs to accelerate water and nutrient transport within plants, thereby augmenting root morpho-physiological traits [14]. Furthermore, Yan et al. [26] observed that Si enhanced the water absorption by increasing the total length and surface area of roots. Maghsoudi et al. [27] suggested that the mechanisms aiding leaf chlorophyll biosynthesis and root growth may be associated with improved nutrient and water uptake in plants. Alharbi et al. [14] found that application of SiO_2_ NPs showed significant potential in ameliorating salinity stress, not only by enhancing the biosynthesis of photosynthetic pigments but also by improving physiological processes like stomatal conductance and relative water content, while reducing electrolyte leakage and proline content in saline-alkali soils. Yan et al. [28] also showed that Si mitigates oxidative damage by modulating the activity of antioxidant enzymes such as SOD, CAT, POD, and ascorbate peroxidase (APX). Yan et al. [26] found that the application of Si significantly increased the activities of SOD, CAT, and APX in rice. Badawy et al. [11] reported that the application of SiO_2_ NPs confers substantial benefits in enhancing ion selectivity by reducing Na^+^ absorption and increasing K^+^ absorption. Khan et al. [29] demonstrated that foliar application of SiO_2_ NPs enhanced the cellular elongation and ion selectivity, while mitigating the detrimental effects of Na^+^ and improving plant growth in saline-alkali soils. Furthermore, deposition of Si in the roots reduces apoplastic bypass flow and provides binding sites for metals ions, resulting in decreased uptake and translocation of Na^+^ from the roots to the shoots [30]. Yan et al. [31] found that silica reduces the net Na^+^ absorption rate, possibly due to the extracellular blocking effect of Si on Na transport, thus lowering the total Na^+^ accumulation in plants [32,33].

The use of SiO_2_ NPs can not only increase the K^+^ content in leaves, but it is also an effective method in agronomy to increase the concentration of available P. For example, Akhtar et al. [34] found that SiO_2_ NPs application improves the soil nutrient content, such as N, P, K, and Si, required for normal plant growth. It has been demonstrated in previous studies that the application of Si increases the availability of P, with positive effects on the uptake and utilization of P observed in rice, wheat, and other crops [35,36,37]. The Si application may affect the mechanisms related to P uptake in plants, such as promoting root exudation of organic acids, thereby mobilizing P in the rhizosphere [38]. Furthermore, silicate anions compete for the same binding sites as phosphate anions, resulting in the release of P into soil solutions and an increase in the P available to plants [39,40]. Akca et al. [41] demonstrated that the application of nano-silica in conjunction with phosphate fertilizer not only enhanced the P content and utilization rate of the fertilizer in crops, but also reduced the quantity of P fertilizer required. The SiO_2_ NPs improve the translocation of N, P, K, and Si from leaves to grains to support grain formation [15,42]. Therefore, the mechanism of reducing salt stress by increasing P content of nano-silicon oxide should be further studied in the future.

Previous studies demonstrated that the application of Si can enhance the quality and nutritional value of rice grains [43,44,45]. However, research on the impact of SiO_2_ NPs on the quality of rice under higher saline conditions is relatively scarce. Our results indicated that the application of SiO_2_ NPs improved the processing and appearance quality as well as the cooking and taste quality of rice. Salt stress inhibits the supply of nutrients, especially during the grain filling stage, and limits the transport of photosynthate partitioning into the grains, leading to a loose arrangement of starch granules within the endosperm that results in the formation of cavities and chalkiness in rice grains [46,47]. This study also showed that the application of SiO_2_ NPs can reduce grain chalkiness, with improved milling and head rice yield. Our results are consistent with Lanning et al. [48], who reported that the starch granules in the chalky areas of rice grains exhibited a blocky or granular structure with porous and loose arrangement characteristics that caused a reduction in the toughness and milling quality of the rice grains.

The cooking and eating quality attributes of rice are deeply influenced by its starch components, i.e., amylose and amylopectin, as well as the presence of structural and functional proteins [7]. It is commonly observed that the higher the concentration of amylose and protein, the higher the viscosity of rice, as amylose and protein can enhance the thermal stability of the starch crystalline matrix, thereby limiting gelatinization and solubilization during cooking. Additionally, an increase in protein concentration can hinder the absorption of water by starch granules, which adversely affects the rice flavor [49]. In our study, the application of SiO_2_ NPs significantly increased the levels of total starch and amylose, while reducing the protein content, hence, improving the taste value of the rice. On the other hand, a high gel consistency is conducive to improving the viscosity and hardness of rice, thereby enhancing the taste value. The quality of rice when cooked and consumed is closely related to the RVA profile of the starch. Typically, a superior taste is indicated by higher PV and BD contrasted with a lower SB. Jin et al. [18] found a significant reduction in PV and BD under salinity compared with CK. Conversely, in our study, the application of SiO_2_ NPs treatment led to a marked increase in both PV and BD, which are important for the culinary taste quality.

The complex structure of amylopectin and its chain length distribution play a key role in the formation of rice cooking and taste [47]. According to Yao et al. [22], saline conditions can reconfigure the starch composition in rice, especially by changing the distribution pattern of amylose and amylopectin chain lengths. Notably, salt-tolerant rice varieties exhibit an increase in intermediate and extended chains under saline conditions, which typically promotes the formation of a more robust double helix conformation, affecting the crystallinity and gelatinization characteristics of starch. It has been observed that saline stress is also associated with a reduction in the proportion of short chains (designated as A chains and B1 chains) while promoting the prevalence of extended chains (B2 chains and B3 chains) [18]. Yao et al. [22] suggested that the increase in gelatinization temperature may be related to the reduction in the number of amylopectin short chains as well as an increase in intermediate and long chains. Additionally, the enthalpy value is positively correlated with gelatinization temperature and crystallinity [2]. Our study also showed that the application of SiO_2_ NPs led to a decrease in gelatinization temperature, and the reduction in crystallinity is mainly due to the decrease in extended chains (B2 + B3) in amylopectin. These shifts are supposed to have repercussions on the crystalline configuration and the overall stability of the starch, which may subsequently alter the sensory attributes and mouthfeel of the rice.

## 5. Conclusions

The SiO_2_ NPs (30 ± 5 nm) application significantly increased salt-tolerant rice yield through enhancing the number of grains per panicle and the rate of grain filling. The application of SiO_2_ NPs markedly boosted chlorophyll levels, leaf area index, potassium ion content, dry matter, root system development, and antioxidant activities and lowered the malondialdehyde content. Additionally, the SiO_2_ NPs treatment improved rice quality by increasing starch and amylose content, enhancing RVA characteristics such as peak viscosity, and reducing crystallinity and gelatinization temperature due to changes in amylose chain length. Overall, SiO_2_ NPs exhibited significant potential in mitigating salinity stress and improving rice growth and quality.

## Figures and Tables

**Table 1:** Effects of SiO_2_ NPs application on rice yield and its components under saline conditions.

Varieties	Treatments	Productive	Grains	Grain	Thousand-Grain	Grain
		Panicle	per	Filling	Weight	Yield
		(m^−2^)	Panicle	(%)	(g)	(t ha^−1^)
YLY957	CK	175.1 ± 10.3 a	135.2 ± 7.7 b	62.5 ± 1.4 b	15.9 ± 0.1 a	2.4 ± 0.3 b
	SiO_2_ NPs	180.3 ± 12.0 a	149.5 ± 3.8 a	67.6 ± 3.2 a	16.1 ± 0.1 a	3.2 ± 0.4 a
JLY534	CK	182.7 ± 6.6 a	143.0 ± 7.7 b	64.9 ± 1.6 b	16.1 ± 0.2 a	3.0 ± 0.4 b
	SiO_2_ NPs	189.6 ± 14.2 a	158.9 ± 4.8 a	69.8 ± 4.8 a	16.2 ± 0.2 a	3.7 ± 0.4 a

Values ± SD (n = 3) in the same column of the same cultivar with different letters are significantly different (*p* < 0.05). YLY957, Y liangyou957; JLY534, Jingliangyou534; CK, control; SiO_2_ NPs, silica nanoparticles.

**Table 2:** Effect of SiO_2_ NPs application on processing and appearance quality of rice under higher saline conditions.

Varieties	Treatment	Brown	Milled	Head	Chalkiness	Chalky	Length/
		Rice	Rice	Rice		Grain	Width
		Rate (%)	Rate (%)	Rate (%)	Rate (%)	Rate (%)	Ratio
YLY957	CK	77.6 ± 0.4 b	68.4 ± 0.8 b	54.9 ± 1.1 b	5.9 ± 0.6 a	15.0 ± 1.7 a	2.9 ± 0.2 a
	SiO_2_ NPs	79.3 ± 0.3 a	70.1 ± 0.3 a	56.2 ± 0.9 a	5.5 ± 0.7 a	13.7 ± 1.2 a	3.0 ± 0.2 a
JLY534	CK	78.4 ± 1.4 a	70.6 ± 0.6 b	57.3 ± 1.2 b	5.9 ± 0.5 a	13.7 ± 0.6 a	2.8 ± 0.1 a
	SiO_2_ NPs	79.2 ± 0.3 a	72.1 ± 0.5 a	59.1 ± 1.2 a	5.5 ± 0.3 a	13.3 ± 0.6 a	3.0 ± 0.2 a

Values ± SD (n = 3) in the same column of the same cultivar with different letters are significantly different (*p* < 0.05). YLY957, Y liangyou 957; JLY534, Jingliangyou 534; CK, control; SiO_2_ NPs, silica nanoparticles.

**Table 3:** Effects of SiO_2_ NPs application on nutritional and eating quality of rice under higher saline conditions.

Varieties	Treatment	Total Starch	Amylose	Protein	Gel	Alkali	Taste
		Content	Content	Content	Consistency	Spreading	Value
		(%)	(%)	(%)	(mm)	Value	
YLY957	CK	70.4 ± 0.5 b	12.8 ± 0.6 b	10.8 ± 0.1 a	66.7 ± 5.9 b	4.1 ± 0.3 b	59.0 ± 1.0 b
	SiO_2_ NPs	72.7 ± 0.8 a	14.7 ± 0.4 a	9.9 ± 0.4 b	73.0 ± 6.2 a	4.8 ± 1.0 a	67.6 ± 1.7 a
JLY534	CK	70.5 ± 1.0 b	13.6 ± 0.2 b	10.8 ± 0.1 a	67.7 ± 0.6 b	6.6 ± 0.3 b	59.2 ± 0.4 b
	SiO_2_ NPs	74.2 ± 1.0 a	17.3 ± 0.6 a	9.5 ± 0.6 b	72.3 ± 2.1 a	6.6 ± 0.3 a	64.6 ± 0.2 a

Values ± SD (n = 3) in the same column of the same cultivar with different letters are significantly different (*p* < 0.05). YLY957, Y liangyou 957; JLY534, Jingliangyou 534; CK, control; SiO_2_ NPs, silica nanoparticles.

**Table 4:** Effects of SiO_2_ NPs application on starch pasting properties under higher saline conditions.

Varieties	Treatment	PV (cP)	TV (cP)	CPV (cP)	BD (cP)	CSV (cP)	SB (cP)	PT (°C)
YLY957	CK	2538.3 b	1737.3 b	2804.0 a	801.0 b	1066.7 a	265.7 b	88.1 a
	SiO_2_ NPs	2878.5 a	1912.0 a	2753.5 b	966.5 a	841.5 b	−125.0 a	87.9 a
JLY534	CK	2872.3 b	1928.3 b	3222.3 a	798.0 a	1294.0 a	350.0 b	87.8 b
	SiO_2_ NPs	2902.3 a	2104.3 a	3234.3 a	944.0 b	1130.0 b	332.0 a	89.6 a

Values ± SD (n = 3) in the same column of the same cultivar with different letters are significantly different (*p* < 0.05). YLY957, Y liangyou 957; JLY534, Jingliangyou 534; CK, control; SiO_2_ NPs, silica nanoparticles; BD, breakdown viscosity; CPV, final viscosity; PT, pasting temperature; PV, peak viscosity; SB, setback viscosity; TV, trough viscosity.

**Table 5:** Effects of SiO_2_ NPs application on starch gelatinization properties under higher saline conditions.

Varieties	Treatment	Onset	Peak	Conclusion	Enthalpy
		Temperature	Temperature	Temperature	(J/g)
		(°C)	(°C)	(°C)	
YLY957	CK	78.2 ± 0.8 a	84.9 ± 0.2 a	89.1 ± 0.2 a	7.7 ± 0.9 a
	SiO_2_ NPs	77.6 ± 0.6 a	83.8 ± 1.5 a	89.4 ± 0.5 a	7.1 ± 0.4 a
JLY534	CK	71.1 ± 0.4 a	76.5 ± 0.6 a	81.5 ± 0.6 a	8.1 ± 0.3 a
	SiO_2_ NPs	69.4 ± 0.5 b	75.2 ± 0.5 b	77.4 ± 6.4 b	7.1 ± 0.2 b

Values ± SD (n = 3) in the same column of the same cultivar with different letters are significantly different (*p* < 0.05). YLY957, Y liangyou 957; JLY534, Jingliangyou 534; CK, control; SiO_2_ NPs, silica nanoparticles.

**Table 6:** Effects of SiO_2_ NPs application on starch amylopectin chain-length distribution and degree of crystallinity under higher saline conditions.

Varieties	Treatment	Relative	Crystal	DP 6–12	DP 13–24	DP 25–36	DP ≥ 37
		Crystallinity (%)	Pattern	(%)	(%)	(%)	(%)
YLY957	CK	21.5 ± 0.4 a	A	25.4 ± 3.6 b	48.6 ± 3.3 a	12.7 ± 0.2 a	13.2 ± 0.7 a
	SiO_2_ NPs	19.7 ± 1.9 b	A	27.6 ± 0.9 a	49.6 ± 0.9 a	11.3 ± 0.1 b	11.5 ± 0.1 b
JLY534	CK	21.2 ± 0.1 a	A	26.4 ± 2.6 a	48.0 ± 1.9 a	12.6 ± 0.4 a	13.0 ± 0.4 a
	SiO_2_ NPs	19.6 ± 0.9 b	A	27.4 ± 0.4 a	49.6 ± 0.3 a	11.2 ± 0.4 b	11.8 ± 0.2 b

Values ± SD (n = 3) in the same column of the same cultivar with different letters are significantly different (*p* < 0.05). YLY957, Y liangyou 957; JLY534, Jingliangyou 534; CK, control; SiO_2_ NPs, silica nanoparticles. The DP 6–12, DP 13–24, DP 25–36, and DP ≥ 37 represent the A chain, B1 chain, B2 chain, and B3 chain, respectively.

## Data Availability

Dataset available on request from the authors.
